# Impact of Probiotics and Prebiotics on Gut Microbiome and Hormonal Regulation

**DOI:** 10.3390/gidisord6040056

**Published:** 2024-09-27

**Authors:** Jelina Basnet, Manar A. Eissa, Licy L. Yanes Cardozo, Damian G. Romero, Samar Rezq

**Affiliations:** 1 Department of Pharmacology and Toxicology, University of Mississippi Medical Center, Jackson, MS 39216, USA; 2 Mississippi Center of Excellence in Perinatal Research, University of Mississippi Medical Center, Jackson, MS 39216, USA; 3 Women’s Health Research Center, University of Mississippi Medical Center, Jackson, MS 39216, USA; 4 Cardiovascular-Renal Research Center, University of Mississippi Medical Center, Jackson, MS 39216, USA; 5 Department of Medicine, University of Mississippi Medical Center, 2500 N. State Street, Jackson, MS 39216, USA

**Keywords:** dysbiosis, probiotics, prebiotics, synbiotics, hormonal disorders

## Abstract

The gut microbiome plays a crucial role in human health by influencing various physiological functions through complex interactions with the endocrine system. These interactions involve the production of metabolites, signaling molecules, and direct communication with endocrine cells, which modulate hormone secretion and activity. As a result, the microbiome can exert neuroendocrine effects and contribute to metabolic regulation, adiposity, and appetite control. Additionally, the gut microbiome influences reproductive health by altering levels of sex hormones such as estrogen and testosterone, potentially contributing to conditions like polycystic ovary syndrome (PCOS) and hypogonadism. Given these roles, targeting the gut microbiome offers researchers and clinicians novel opportunities to improve overall health and well-being. Probiotics, such as *Lactobacillus* and *Bifidobacterium*, are live beneficial microbes that help maintain gut health by balancing the microbiota. Prebiotics, non-digestible fibers, nourish these beneficial bacteria, promoting their growth and activity. When combined, probiotics and prebiotics form synbiotics, which work synergistically to enhance the gut microbiota balance and improve metabolic, immune, and hormonal health. This integrated approach shows promising potential for managing conditions related to hormonal imbalances, though further research is needed to fully understand their specific mechanisms and therapeutic potential.

## 1. Introduction

The human body is home to a vast array of microorganisms that reside in various areas, such as the gut, skin, mouth, and vagina, with the gastrointestinal (GI) tract being the primary site [1]. This collection of microorganisms forms the microbiome, which is composed of various microbial species known collectively as the microbiota [2]. The gut microbiota plays a critical role in human health, interacting closely with the endocrine system through mechanisms that involve hormones regulating host behavior, metabolism, immunity, insulin signaling, and other functions [3-5]. The gut microbiota can influence host behavior by modulating neurohormones like serotonin, dopamine, and gamma-aminobutyric acid (GABA), as well as stress hormones like cortisol [6].

The gut microbiota is increasingly being shown to influence the host immune net-work [7,8]. This relationship begins at birth, with the microbiota playing a crucial role in shaping immune system development, which in turn impacts the composition of the microbiome [7]. The gut microbiota also significantly affects the host’s metabolic status through multiple mechanisms. It produces short-chain fatty acids (SCFAs) like butyrate, propionate, and acetate. SCFAs via G protein-coupled receptor 43 (GPR43) activation can reduce inflammation and lipolysis, increase adipogenesis and leptin release, and ultimately lead to lower fat accumulation [9]. Additionally, SCFAs activate AMP kinase in muscles, which reduces lipid accumulation and improves insulin sensitivity [9], aiding in appetite regulation and weight management [9,10]. Furthermore, the gut microbiota produces metabolites that act as signaling molecules, influencing the release of key metabolically active hormones such as serotonin, glucagon-like peptide-1 (GLP-1), peptide YY (PYY), and cholecystokinin (CKK) from enteroendocrine cells (EC) in the gut [3]. These hormones regulate important metabolic processes, including glucose metabolism, insulin sensitivity, adiposity, and appetite [3]. SCFAs, in particular, are known to modulate the secretion of these gut peptides [11]. GLP-1 and PYY, secreted by L-cells located primarily in the ileum and colon, play essential roles in regulating food intake and satiety. The gut microbiota’s influence on GLP-1 and PYY secretion highlights its significant implications for the development of metabolic diseases [3]. CCK, secreted from “I cells” predominantly found in the upper small intestine [12], is released in response to dietary fat and protein intake. However, the regulation of CCK by gut microbes is less well understood due to the limited exposure of CCK-containing cells to the microbiota in the small intestine [3].

Recent evidence highlights a possible significant role of the gut microbiota in regulating sex steroid levels. It affects estrogen metabolism through the estrobolome, a collection of bacterial genes encoding enzymes such as β-glucuronidase [13,14]. These enzymes deconjugate estrogens, impacting their bioavailability and circulating levels [14]. Additionally, the gut microbiota has been identified as a major regulator of androgen metabolism in the intestines, leading to relatively high levels of free dihydrotestosterone (DHT), the most potent androgen, in the colonic contents of young and healthy mice and men [15].

A disrupted gut microbiome, in turn, can have detrimental effects on reproductive and metabolic health through hormonal fluctuations and inflammation. This imbalance in the gut microbiota can lead to altered sex hormone levels and metabolic dysfunctions, contributing to conditions such as PCOS, infertility, and various metabolic disorders [13,16-18].

## 2. Dysbiosis as a Possible Trigger of Hormonal Disorders

**Figure 1.**
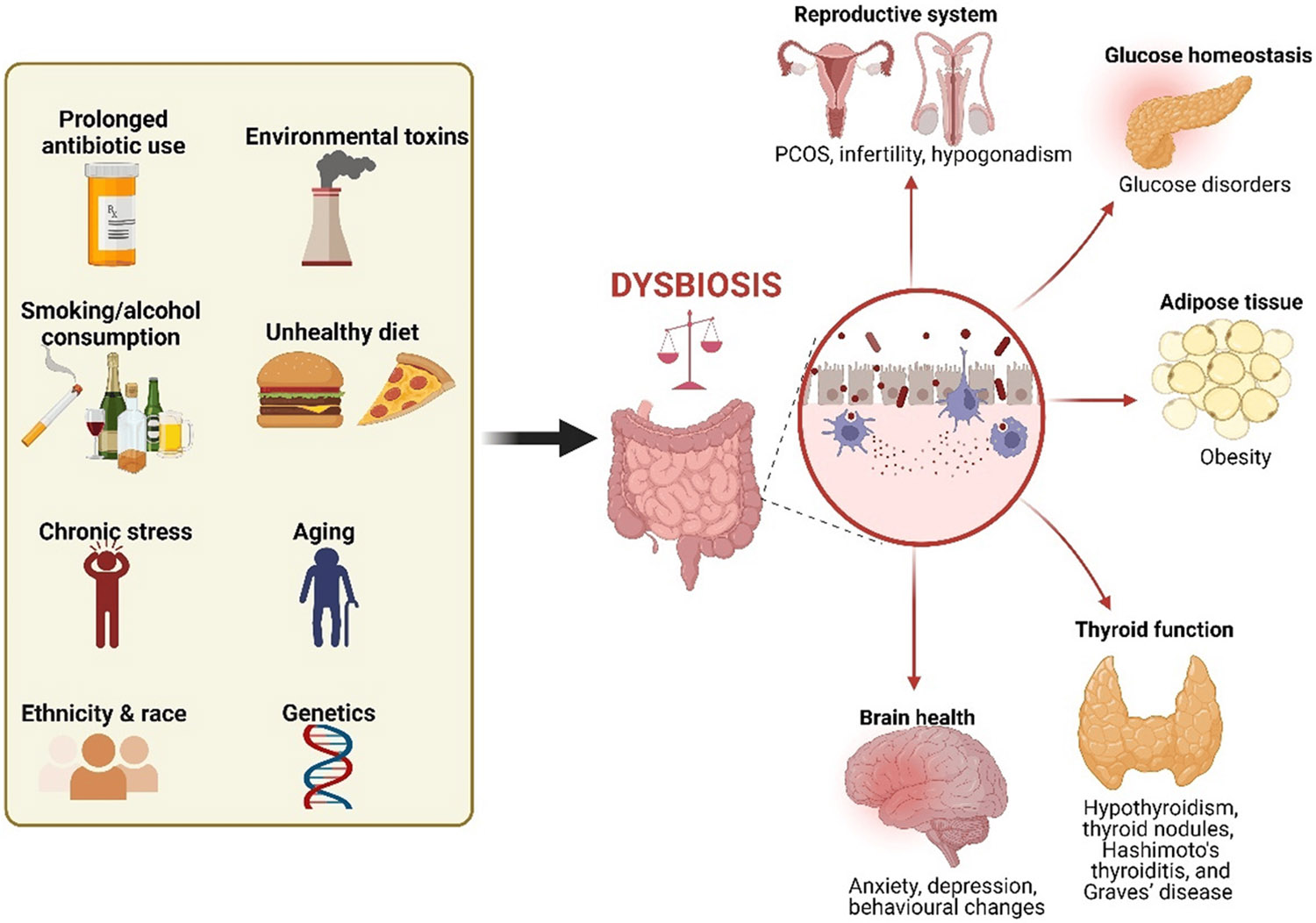
Factors that can induce dysbiosis and the link to hormonal disorders. Dysbiosis can result from factors such as xenobiotics, poor lifestyle habits, chronic stress, environmental toxins, age, ethnicity, and genetics. This imbalance can lead to hormonal fluctuations and inflammation, contributing to several reproductive and metabolic disorders. Created in BioRender.

Gut dysbiosis refers to an imbalance or disruption in the composition and function of the gut microbiome. Dysbiosis can be triggered by various factors, including xenobiotics (such as prolonged antibiotic use), lifestyle habits (such as an unhealthy diet, smoking, and alcohol use), health status (such as chronic stress, infections, and chronic conditions like inflammatory bowel disease; IBD and irritable bowel syndrome; IBS), as well as environmental toxins, age, ethnicity, and genetic background [13,19,20] (Figure 1).

Gut dysbiosis can lead to alterations in the production and signaling of neurotransmitters and hormones, such as serotonin, dopamine, and cortisol [21]. These imbalances can have profound effects on mood, cognition, and overall brain function [22]. Individuals with gut dysbiosis are more likely to experience symptoms of depression and anxiety [23]. A study in germ-free (GF) mice and specific pathogen-free (SPF) mice showed that altering microbial colonization can affect behavioral responses to chronic stress by modulating hormones and hormone receptors in the hypothalamic–pituitary–adrenal (HPA) axis under stress [24]. Additionally, the altering gut microbiome can influence the production of neurotrophic factors, which are essential for brain development and repair [25].

Recent studies suggest a possible link between gut dysbiosis and female hormonal disorders [20]. Dysbiosis can lead to fluctuations in circulating estrogens by altering β-glucuronidase activity, which may contribute to metabolic complications, PCOS, and female infertility [13,16]. Additionally, dysbiosis may promote PCOS development, the most common reproductive endocrine disorder in females, by increasing gut permeability, leading to systemic inflammation and insulin resistance (IR) [26]. Dysbiosis-induced hypoestrogenemia could also influence the progression of endometriosis and its potential malignant transformation [27]. Hypoandrogenism in males and hyperandrogenism in females are both types of androgen disorders. Gut microbial imbalance can contribute to androgen synthesis dysfunction, which may lead to androgen-driven diseases such as obesity, metabolic syndrome, PCOS in females, and male hypogonadism [15,28].

Intestinal dysbiosis may affect the secretion of multiple hormones and vitamins, including vitamin D, thyroid hormones, and insulin [29,30]. Recent evidence indicates that primary hypothyroidism is associated with altered bacterial diversity and reduced SCFA production, which may contribute to thyroid dysfunction by lowering thyroxine levels [31]. There is also an established link between the gut microbiome and other thyroid disorders, such as thyroid nodules, Hashimoto’s thyroiditis, and Graves’ disease [32-35].

A healthy gut microbiome is essential for maintaining glucose homeostasis. Evidence from basic and clinical studies indicates that gut dysbiosis can be a causal or contributing factor in the pathogenesis of various glucose metabolism disorders, including obesity, IR, and Type-1 and Type-2 Diabetes [36]. Turnbaugh and colleagues demonstrated that gut microbiota dysbiosis can cause metabolic disease in mice independent of genetic background [37]. Their study showed that microbiota transplantation from mice with diet-induced obesity to lean germ-free mice recipients resulted in more fat deposition than transplants from lean mice donors. Likewise, another study reported that the transplant of microbiota from lean and obese human twins into germ-free mice lacking a native gut microbiome resulted in the conveyance of the metabolic phenotype of the host [38]. These findings highlight the potential of targeting the gut microbiome as a strategy for preventing and treating disorders related to hormonal imbalances. Common approaches include the use of probiotics, prebiotics, and synbiotics.

### 3. Probiotics: Definition, Mechanisms of Action, and Impact

Probiotics are living, non-pathogenic microorganisms that offer benefits to human health when consumed in adequate amounts [2]. Multiple microorganisms that belong to the genera *Propionibacterium, Lactococcus, Enterococcus, Pediococcus,* and *Bacillus* are considered to be probiotics [39]. Still, the most important probiotic strains are the *Lactobacillus* and *Bifidobacterium*, which are commonly used in functional foods and dietary supplements [40,41]. While microbiota refers to the natural population of microorganisms in the body, probiotics are beneficial microbes that are taken to support or enhance the microbiota.

Probiotics have been found to play a supportive role in the treatment and prevention of various conditions, including IBD, IBS, lactose intolerance, cancer, diarrhea, and allergic diseases [39]. The major therapeutic effects of probiotics are primarily attributed to their direct or indirect effect on the GI tract [42]. These beneficial effects are attributed to several key mechanisms by which probiotics eradicate pathogens and maintain a healthy balance of gut flora. These mechanisms include competing with pathogens for nutrients and adhesion sites in the gut, enhancing intestinal barrier functions, improving the immune system, and producing neurotransmitters [39], which makes it difficult for harmful pathogens to thrive. Probiotics also function as antimicrobial agents by producing substances, such as organic acids and hydrogen peroxide, which combat the pathogenic bacteria in the gut [43]. In addition, probiotics increase the production of mucin proteins, which strengthen the function of the intestinal barrier [44].

Apart from the direct effect on the GI tract, the gut microbiome interacts with the body’s endocrine system via several complex mechanisms. One important pathway is the gut–brain axis [45]. Probiotics influence the production and release of a number of neurotransmitters and hormones, such as dopamine, serotonin, and norepinephrine [46]. Additionally, probiotics reduce the level of stress hormones such as cortisol [47,48]. Accordingly, probiotics have a role in regulating depression, anxiety, and other central nervous system (CNS)-related disorders [48,49]. While existing studies highlighted the impact of probiotics on neurotransmitter production, stress hormone levels, and CNS-related disorders, further research is warranted to determine the strain-specific effects of probiotics for targeting specific CNS disorders.

The synthesis of GI hormones such as leptin, ghrelin, and GLP-1 is influenced by specific strains of microbiota, indicating a role for the microbiota in appetite regulation [3,50]. Probiotics contribute to the fermentation of dietary fibers, producing SCFAs that positively affect metabolism and enhance the release of hormones involved in appetite control and insulin secretion [41,51]. While the impact of probiotics on GI hormones is well documented, the precise mechanisms through which different probiotic strains modulate these hormones remain unclear and warrant further research.

In a recent observational study, probiotic use was associated with higher estradiol levels in premenopausal women and lower total testosterone levels among pre- and postmenopausal women [52]. In ovariectomized mice, probiotics were found to influence estrogen levels by modulating the gut microbiota, enhancing SCFA production, and up-regulating estrogen receptors in adipose tissue [53]. While the gut microbiota’s role as a major regulator of colonic androgen content in young and healthy mice and men is well documented [15], there is a lack of studies investigating the impact of probiotics on androgen regulation in both males and females. Moreover, despite the potential benefits of probiotics for overall health in both men and women, their role in preventing or treating sex hormone-related disorders, such as hypogonadism in men and PCOS in women, remains understudied.

## 4. Prebiotics: Definition, Mechanisms of Action, and Impact

While probiotics are live beneficial bacteria that support gut health, prebiotics are non-digestible fiber compounds that selectively nourish the gut microbiota, stimulating their growth and activity. This selective stimulation of the microbiota ultimately confers health benefits to the host [54]. Importantly, a prebiotic must be resistant to stomach acid, remain unabsorbed in the GI tract, be fermented by microbiota, and selectively stimulate the growth and activity of beneficial intestinal bacteria [55]. Prebiotics include diverse carbohydrates, including fructans, β-glucans, galacto-oligosaccharides, inulin, starch, guar gum, lactulose, maltodextrin, xylo-oligosaccharides, and arabino-oligosaccharides [54,56].

By promoting a healthy gut microbiome, prebiotics contribute to improving physical health. Several studies have reported the positive role of prebiotics on the GI tract. For instance, prebiotics can help manage conditions like bloating and constipation [57]. In a randomized controlled trial involving patients with functional bowel disorders, the administration of fructo-oligosaccharides (FOSs) over a six-week period was found to improve the symptoms of IBS [58]. FOS supplementation was also shown to decrease Crohn’s disease activity in patients in a clinical trial [59]. Prebiotic fermentation products have also demonstrated protective effects against the development and progression of colorectal cancer [60,61]. In addition, prebiotics have been found to aid in weight management in both adults and children [62,63]. Because the GI tract is connected to the brain via the gut–brain axis, prebiotics have positive effects on the nervous system, such as improved cognition and memory [64].

Prebiotics offer numerous health benefits by selectively stimulating the growth and activity of beneficial bacteria in the gut. Their primary mechanism of action involves promoting the growth of beneficial bacterial strains like *Lactobacilli* and *Bifidobacteria*, which outcompete pathogenic microbes for resources and attachment sites, thereby enhancing gut health [65]. Additionally, the fermentation of prebiotics produces SCFAs, which diffuse through gut enterocytes and enter blood circulation, affecting not only the GI tract but also distant organs and systems [66]. The acids produced from prebiotic fermentation alter the gut environment by decreasing its pH, leading to changes in the composition and population of gut microbiota [67]. Prebiotics also improve gut barrier function by increasing mucin production and strengthening the tight junctions between intestinal cells, which helps prevent harmful substances from entering the bloodstream [68,69]. Furthermore, prebiotics stimulate the immune system by increasing the population of beneficial microbes in the gut and altering cytokine expression [70].

The prebiotic, inulin, has been shown to increase plasma levels of GLP-1 and reduce levels of ghrelin [71,72]. This suggests that prebiotics can influence GI hormone production, likely through the production of SCFAs, thereby affecting appetite regulation. As a result, prebiotics could serve as new targets for managing obesity and other eating disorders. In addition, prebiotics may help people cope with stress and mild anxiety by lowering cortisol levels, a stress hormone [73]. Some prebiotics have also been reported to increase estrogen metabolism in the intestine by suppressing β-glucuronidase activity [74], which could potentially reduce the risk of estrogen-mediated cancers. However, data on the role of prebiotics in hormone regulation are still limited. More research is needed to fully understand their impact on hormonal regulation and their potential therapeutic uses.

## 5. Synergistic Effects of Probiotics and Prebiotics (Synbiotics)

Synbiotics are a specific combination of probiotics, microorganisms that provide health benefits when consumed, and prebiotics, compounds that promote its growth, having a synergistic effect when paired together [75,76]. In May 2019, the International Scientific Association for Probiotics and Prebiotics (ISAPP) updated the definition of a synbiotic to “a mixture of live microorganisms and substrate(s) that confer health benefits to the host” [77]. Synbiotics are classified mainly into two groups: (a) complementary synbiotics and (b) synergistic synbiotics [75]. The complementary synbiotics are composed of probiotics and prebiotics that provide health benefits independently of each other, without requiring any mutual function. In contrast, synergistic synbiotics include a substrate that is specifically utilized by the co-administered live microbial populations, enhancing their effectiveness [75,77].

The use of synbiotics is an efficient and promising approach for maintaining gut microbiota homeostasis, promoting the restoration and maintenance of beneficial gut bacteria [78]. A randomized controlled trial has demonstrated that synbiotics can significantly improve metabolic health in individuals with metabolic syndrome and prediabetes [79]. Synbiotic supplementation under high-fat diet conditions has been found to alleviate metabolic disturbances and improve intestinal barrier integrity by increasing gut hormones and SCFAs [80]. Some potential benefits of synbiotic consumption in humans include the following: (a) increasing the populations of *Lactobacilli* and *Bifidobacterial*, which helps maintain gut microbiota balance; (b) boosting the production of SCFAs; (c) improving metabolic processes such as bile acid deconjugation and mineral absorption; (d) strengthening the modulation of the host immune system; and (e) enhancing liver function in individuals with cirrhosis and other [75,81-83]. Overall, the synbiotic approach has proven to be more effective than using prebiotics or probiotics alone in modulating gut microbiota and alleviating metabolic disorders associated with an imbalanced gut microbiota in humans [84].

## 6. Probiotics and Prebiotics in the Management of Endocrine Disorders

The potential role of probiotics in hormonal regulation and the management of endocrine disorders is suggested by recent findings from both basic and clinical research (Table 1). A recent observational cohort study among 2699 women, comprising a nationally representative sample of adults who participated in the National Health and Nutrition Examination Survey between 2013 and 2016, suggests the potential beneficial effect of probiotics. Probiotic ingestion was considered when a subject reported yogurt or probiotic supplement consumption. The data revealed that premenopausal women who consumed probiotics had higher estradiol levels, and postmenopausal women who consumed probiotics had lower total testosterone levels than women who did not consume probiotics [52]. Whether these findings could be extrapolated to other clinical conditions is unclear. For example, among patients with type 2 diabetes mellitus (T2DM), women have a higher level of circulating testosterone [85] and could be a population that could greatly benefit from probiotics. Another condition where excess of androgen is present in women is PCOS. About 80% of women with PCOS have hyperandrogenemia, and the level of testosterone is about 1.5-fold higher compared to women with normal cycling. Interestingly, women with PCOS and elevated androgen levels have a worse cardiometabolic profile compared to women with PCOS with normal levels of androgens [86]. Thereby, it can be speculated that a simple intervention such as yogurt or probiotics intake may result in beneficial hormonal changes that will decrease cardiovascular risk in those populations. However, this hypothesis remains to be tested. In contrast, in men with hypogonadism, probiotics administration failed to increase the plasma level of testosterone [87]. Whether and how probiotics, in a sexually dimorphic manner, regulate the levels of sex steroids remains to be elucidated.

Early puberty is defined by the development of secondary sexual characteristics and menses before eight years of age in girls and nine years in boys. Early puberty has been extensively linked to adverse health outcomes, such as metabolic syndrome. Recent data suggest that probiotic drinks or yogurt have a protective effect against early puberty [88]. Thereby, probiotics administration could constitute an effective intervention to modulate sex steroids in a variety range of clinical conditions.

Besides sex steroids, other steroids, such as cortisol, could also be impacted by prebiotics. Cortisol, or the stress hormone, has several functions in the human body, such as mediating the stress response, regulating metabolism, the inflammatory response, and immune function [89]. Data from a small randomized clinical trial demonstrated that a three-week consumption of two types of prebiotic supplements in healthy human volunteers was associated with decreased waking salivary cortisol reactivity (a stress biomarker) and improvement in anxiety [90].

The thyroid hormones are well known for controlling metabolism, growth, and many other critical functions. Recent data have suggested that microbes influence thyroid hormone levels by regulating iodine uptake, degradation, and enterohepatic cycling [35]. A recent meta-analysis of eight randomized clinical trials has shown that although probiotics and prebiotics did not change the level of thyroid hormones, they may modestly reduce thyroid-stimulating hormone receptor antibody levels in patients with hyperthyroidism [91].

T2DM, a metabolic disorder characterized by elevated glucose levels, has emerged as a major public health problem. Its prevalence is increasing, and it is estimated that by 2045, 700 million individuals worldwide are expected to have diabetes mellitus. A recent meta-analysis of 22 randomized clinical trials, including a total of 2218 patients, suggested that probiotics may lower baseline levels of HbA1c, fasting glucose, and IR in patients with T2DM. Similar findings were observed in women with gestational diabetes [92]. Metformin is an antihyperglycemic medication approved for the management of T2DM when glycemic control cannot be accomplished by lifestyle modification alone. Metformin is also recommended for diabetes prevention in patients age < 60 years and/or BMI ≥ 35 kg/m^2^, or HbA1c of 5.7% to 6.4%, in whom lifestyle modifications failed to reduce hyperglycemia Metformin is the initial therapy of choice in T2DM due to its efficacy, weight-neutral effect, general tolerability, favorable cost, and protection from cardiovascular events [93]. Recent data demonstrated that the co-administration of oral probiotic interventions along with metformin treatment was found to significantly improve glycemic control in T2DM patients [94].

Dyslipidemias, or abnormal levels of cholesterol and/or triglycerides, are frequently associated with T2DM. The administration of probiotics is associated with improvement in the lipid profile of patients with dyslipidemias [95]. However, whether this improvement of glycemic parameters or lipids results in an improvement in cardiovascular morbidity or mortality remains unknown.

## 7. Fecal Microbiota Transplantation

Fecal microbiota transplantation (FMT) refers to administering stool bacteria into the intestinal tract of a patient, a clinically relevant example is the treatment of recurrent Clostridioides difficile infection (CDI) [103]. Patients with recurrent CDI have a reduced diversity and number of the intestinal microbiome compared to healthy individuals [104]. This infection can be observed in up to 20% of antibiotic users. The mechanism is not entirely understood, but it is related to changes in the homeostatic balance of the GI mucosa. The alteration of the colonic microbiota following FMT appears to be long-term, with a high cure rate after FMT [105]. Limited data suggest that FMT can also be beneficial in CDI-associated bloodstream infections [106]. Additionally, FMT has demonstrated potential in reducing dysbiosis, decreasing hospitalizations, and improving disease severity in patients with hepatic encephalopathy and liver cirrhosis. It has also been shown to enhance metabolic outcomes in patients with non-alcoholic fatty liver disease [107].

The impact of FMT on the endocrine system has been suggested in recent findings in cross-sex fecal transplants in Wistar rats. Male rats that received FMT from female donors displayed lower plasma concentrations of testosterone compared to the male recipients that received same-sex FMT without changes in other hormones such as cortisol [108]. It is very early to fully understand the clinical relevance of these findings and the mechanisms underlying this change. Recent findings suggest the existence of local testosterone synthesis and metabolism in the colon, with higher concentrations than those in plasma in male rats [109]. This finding is not novel per se; several other organs have the full machinery necessary to synthesize or activate sex steroids [110-112]. Although the sex of the donor is accounted for in some transplants, such as the heart [113], it is not usually the case for FMT. Clinical evidence also suggests that FMT may help preserve endogenous insulin production in patients recently diagnosed with type 1 diabetes [114]. However, further research is needed to fully understand the effects of FMT on the endocrine system.

## 8. Limitations, Future Directions, and Research Gaps

Despite their proven benefits to improve gut health overall, the efficacy of probiotics is limited by several factors. For example, patients who have long-term dysbiosis as a consequence of chronic gut inflammatory conditions, such as Crohn’s disease or ulcerative colitis, may be resistant to new colonization introduced by probiotics, reducing their efficacy [115]. Similarly, the concurrent use of antibiotics can also limit probiotic efficacy [116]. Since probiotics rely on fibers as substrates [117], a diet high in sugar and low in fiber creates a poor environment, further reducing their effectiveness. However, this limitation can be mitigated by the use of probiotics or synbiotics. Although generally considered safe for healthy populations, the use of certain probiotic species in immunocompromised, very young, or elderly patients carries the risk of adverse effects, such as fungemia, fungal septicemia, endocarditis, probiotic-associated pneumonia, allergic responses, and abdominal or liver abscesses [118]. Therefore, the effectiveness of probiotics, prebiotics, and synbiotics depends heavily on the specific strains used and the individual’s unique microbiome profile. Using non-personalized or generic strains that do not address specific microbial imbalances may result in suboptimal outcomes. The personalization of treatment, proper microbial balance, and careful consideration of underlying health conditions are crucial for optimizing the benefits of these interventions.

The use of probiotics is not fully established in clinical practice. This is mainly due to the sizeable, significant heterogeneity in the studies and variability in results. Large-scale randomized clinical trials with clear and predefined endpoints are necessary to fully determine the efficacy and safety of probiotics in humans. Also, clear protocols and dose-dependent effects are required to untangle the complex impact of probiotics in chronic conditions. The findings from basic research could be beneficial in describing novel mechanisms and informing about efficacy and safety that could be translated to humans.

## 9. Conclusions

A disrupted gut microbiome can negatively impact reproductive and metabolic health by affecting the hormonal system. Due to its relatively safe profile, probiotic and prebiotic supplementation has drawn considerable interest recently as potential strategies to improve gut health. However, stronger evidence, such as data from large randomized clinical trials in different groups, is needed to better estimate its efficacy and safety. Other caveats are the wide-ranging variations in the composition of the probiotics administered, the dosage and duration of the probiotic interventions, and limited endpoints, which could explain the inconsistent findings across studies. Regardless of the gaps in our knowledge, probiotics and prebiotics are emerging as novel co-adjuvant therapies in treating several endocrine disorders.

## Figures and Tables

**Table 1.** Role of gut microbiome in hormonal regulation.

Hormone of Interest	Main Findings	Proposed Mechanisms	Reference
Cortisol, adrenocorticotropic hormone, (ACTH), aldosterone	Germ-free (GF) mice exhibit an imbalance in the HPA axis, affecting the neuroendocrine system in the brain and resulting in an anxiety-like behavioral phenotype in response to chronic restraint stress.	Tendency to ↑ Cortisol ↑ ACTH ↑ Aldosterone ↑ Corticotropin-releasing hormone receptor 1 (Crhr1) mRNA levels ↓ Mineralocorticoid receptor (MR) mRNA levels	[24]
	Probiotic formulation exerts anxiolytic-like effect in rats and beneficial psychological effects in healthy human volunteers.	↓ Urinary free cortisol in tested subjects	[47]
	Probiotic *Bifidobacterium longum* 1714 reduced stress and improved memory in healthy human volunteers.	↓ Salivary cortisol	[96]
	Prebiotics: FOS and B-GOS intake reduces the waking cortisol response and alters emotional bias in healthy volunteers.	↓ Salivary cortisol	[73]
Estrogen	Gut bacterial species containing β-glucuronidases and β-glucuronides enzymes are capable of metabolizing estrogens.	The deconjugation and conjugation of estrogen by the estrobolome modulate the enterohepatic circulation of estrogens, thereby affecting circulating and excreted estrogen levels	[97]
	In men and postmenopausal women, the level of total urinary estrogens was strongly and directly associated with fecal microbiome richness.	Altering β-glucuronidase activity	[98]
	Dysbiosis may influence the progression of endometriosis in females.	↓ Estrogen level	[27]
	Diet rich in the probiotic *Lactobacillus plantarum* and soy isoflavones reverses menopausal obesity and increases circulating estrogen levels in ovariectomized mice.	↑ Serum estradiol, upregulate estrogen Receptor a (ERα) in adipose tissue. ↑ SCFA production	[53]
	Probiotic supplements and yogurt intake are associated with higher estradiol levels among premenopausal women.	-	[52]
Androgens	Gut microbiota produces high free levels of DHT in the colonic content of young and healthy mice and men.	De-glucuronidation of DHT and testosterone	[52]
	Probiotic supplements and yogurt intake are associated with lower total testosterone levels among postmenopausal women.	-	[52]
Insulin	High-fat diet induces gut dysbiosis, promoting insulin resistance in TLR5-deficient mice.	Disrupting insulin signaling	[99]
	Gut microbiota from obese donors induced insulin resistance in recipient mice.	Altering host gut microbiota composition	[38]
	Gut microbiota alteration can impair insulin signaling and cause insulin resistance.	Increased intestinal permeability, lipopolysaccharide absorption, and inflammatory pathway activation	[100]
	Probiotic VSL#3 I improve metabolic status and insulin sensitivity in overweight adults.	↓ Circulating inflammatory markers and insulin Improves the lipid profile and decreases the atherogenic index	[101]
	Prebiotic oligofructose improve glucose tolerance and glucose-induced insulin secretion in high fat fed mice.	↓ *Bifidobacterium* spp. ↓ Endotoxemia and plasma and adipose tissue proinflammatory cytokines ↑ Colonic mRNA levels of the GLP-1 precursor proglucagon	[102]
Leptin, Ghrelin, GLP-1	Gut microbiota affects the levels/signaling of GI hormones such as leptin, ghrelin, and GLP-1.	SCFAs modulate leptin release via activating GPR41 receptor SCFAs induce GLP-1 release through interacting with enteroendocrine cells SCFAs attenuate ghrelin-mediated signaling via the growth hormone secretagogue receptor-1a lipopolysaccharide (LPS) modulates GLP-1 release via TLR4	[3,50]
	The prebiotics inulin and oligofructose exert favorable effects on glucose and lipid metabolism.	↑ GLP-1 production ↓ Serum ghrelin levels	[71]
Thyroid	Gut dysbiosis negatively impacts the thyroid function in humans.	↓ SCFA production ↓ Thyroxine levels	[31]

## Data Availability

No new data were created or analyzed in this study. Data sharing is not applicable to this article.
